# Early impact of the COVID-19 pandemic and social restrictions on ambulance missions

**DOI:** 10.1093/eurpub/ckab065

**Published:** 2021-04-15

**Authors:** Lauri Laukkanen, Sanna Lahtinen, Janne Liisanantti, Timo Kaakinen, Ari Ehrola, Lasse Raatiniemi

**Affiliations:** 1 Research Group of Surgery, Anaesthesiology and Intensive Care Medicine, Medical Research Center of Oulu University, Oulu University Hospital, Oulu, Finland; 2 Department of Anaesthesiology, Oulu University Hospital, Oulu, Finland; 3 Oulu-Koillismaa Rescue Department, Emergency Medical Services, Oulu, Finland; 4 Centre for Pre-Hospital Emergency Care, Oulu University Hospital, Oulu, Finland

**Keywords:** COVID-19, emergency medical services, prehospital care, social restrictions

## Abstract

**Background:**

The SARS-CoV-2 coronavirus disease (COVID-19) has had a major impact on health care services globally. Recent studies report that emergency departments have experienced a significant decline in the number of admitted patients in the early phase of the pandemic. To date, research regarding the influence of COVID-19 on emergency medical services (EMS) is limited. The present study investigates a change in the number and characteristics of EMS missions in the early phase of the pandemic.

**Methods:**

All EMS missions in the Northern Ostrobothnia region, Finland (population 295 500) between 1 March to 30 June 2020 were screened and analyzed as the study group. A control group was composed from the EMS calls between the corresponding months in the years 2016–2019.

**Results:**

A total of 74 576 EMS missions were screened for the study. Within the first two months after the first COVID-19 cases in the study area, the decline in the number of EMS missions was 5.7% – 13% compared to the control group average. EMS time intervals (emergency call to dispatch, dispatch, en-route, on-scene and hospital handover) prolonged in the COVID-19 period. Dispatches concerning mental health problems increased most in the study period (+1.2%, p < 0.001). Only eleven confirmed COVID-19 infections were encountered by EMS in the study period.

**Conclusion:**

Our findings suggest that the present COVID-19 pandemic and social restrictions lead to changes in the EMS usage. These preliminary findings emphasize the importance of developing new strategies and protocols in response to the oncoming pandemic waves.

## INTRODUCTION

In December 2019, a novel coronavirus (SARS-CoV-2) was identified in Wuhan, China, and it evoked a global pandemic in the spring of 2020 [1]. The coronavirus disease (COVID-19) is characterized by asymptomatic infection to severe pneumonia and respiratory failure[2]. To date (13 March 2021), over 118 million cases and over 2 million fatalities worldwide have been reported so far [1].

A decline in the number of patients presenting to emergency department (ED) has been reported in the early phase of the COVID-19 pandemic [3, 4]. Several efforts in the forms of social restrictions and nationwide lockdowns have been implemented trying to reduce the spread of this disease [5]. In the context of emergency medical services (EMS), there is a limited amount of research concerning the effect of COVID-19. A single study from Switzerland reported an increase in the number of emergency calls and ambulance dispatches in the early weeks of the pandemic. [6] Although, contrary findings have also been reported [7].

The objective of the present study was to examine if there was a change in the number and characteristics of EMS missions and patients in early phase of the COVID-19 pandemic compared to previous years. Further, our aim was to investigate the prevalence of suspected and confirmed COVID-19 cases in EMS patients.

## METHODS

### Study design and ethics

The study design is an observational, retrospective study. The approval was obtained from the administration of Oulu University Hospital (OUH) (Reference number 54/2019, updated on 17th June 2020). According to the local policies and due to the retrospective study design, approval from the ethics committee and consent from the patients were not needed. The STROBE checklist for observational studies was used in reporting this study.

### Study area and COVID-19 spread

The study was carried out in the Northern Ostrobothnia region, in Northern Finland which is a mixed rural-urban region with 295 500 inhabitants and population density of 12.8 per square kilometer. The first

COVID-19 case in the study area occurred on 6 March 2020, followed by a total of 136 confirmed cases during the study period. Correspondingly, 7271 COVID-19 cases occurred in Finland during the same period [8]. In early March 2020, the number of COVID-19 cases increased rapidly in Finland, and health care services could not respond to the rising demand of COVID testing. The Finnish Institute for Health and Welfare (THL) calculated models for the expected progression of the pandemic, and the risk of an overcapacity of ICU's and hospital network was considered as a substantial threat. In response, on 16th March 2020, the Finnish Government declared a state of emergency, forcing various restrictions on Finnish society, including education, public and cultural services, public gatherings and travel [Supplementary Table 1]. The state of emergency was lifted on 16th of July 2020.

**EMS and hospital network**

All emergency calls (EMS, police, and fire) in the study area are responded by the emergency medical communication center (EMCC), where the call is evaluated by an emergency dispatcher. Dispatcher determines mission code and priority and dispatches the corresponding organization. Four classes of priority are used, urgent priorities A (highest) and B, and non-urgent priorities C and D (lowest).

A tiered EMS system includes basic- and advanced life support ambulances (BLS and ALS), field supervisor, and physician-staffed helicopter emergency medical services (HEMS). BLS units are operated by emergency medical technicians (EMTs) or firefighters, and ALS includes at least one paramedic. The local non-transportation guidelines have an option that the EMS crew may refer patients to hospital by other means than ambulance or treat them at the scene. The non-transport rate in the study area is generally around 38% [9]. There is a tertiary level care hospital (OUH) and several smaller municipal health centers providing primary health care services in the study area. During the COVID-19 outbreak, ED in OUH was divided into areas for infectious and non-infectious patients, and few of the smaller health centers in the study area were focused to treat only infectious patients. In EMCC, routinely asked questions about infectious symptoms and COVID-19 exposure were added to the EMCC key-question protocol of EMS calls. In presence of these symptoms or exposure, the dispatcher gave a "coronavirus suspicion" to the responding EMS unit.

**Setting and participants**

All EMS missions in the study area between 1 March and 30 June 2020 were retrospectively screened and were assigned to the study group. According to the Finnish Institute for Health and Welfare (THL) statistics, the first wave of COVID-19 took place during this period, a peak of cases occurring in early April [8]. The control group for comparison comprised EMS missions in the same area between the corresponding months (1 March to 30 June) in the years 2016 – 2019.

**Data collection and variables**

The EMS provider in the study area uses an electronic patient documenting system (Merlot Medi, CGI© Suomi Oy). The Hospital District of Northern Ostrobothnia (PPSHP) has an electronic patient management system (Oberon, Uranus) for administrative registration of hospital treatment, diagnosis, and care pathways. Data concerning the number of missions per day, timeline of the dispatch, mission code, priority, characteristics, and clinical information were retrieved from the EMS database. Information of the suspected or confirmed COVID-19 infection were collected from the hospital district database.

**Statistical analysis**

The statistical analysis was performed using the SPSS statistics software (IBM© Corp., SPSS Statistics for Windows, version 25.0). For the analysis of the change in the number of EMS missions, a mean number of daily EMS missions was calculated for each month. For further analysis, missions within the years 2016 – 2019 were summed to a single control group. The population data including the age groups was retrieved from the open database of statistics Finland. The rates of EMS missions for 1000 inhabitants per year were calculated using the population of the area in the year 2018 as the index year.

Continuous data is presented as means and standard deviations (±SD) or medians and 25$^{th}$ and 75$^{th}$ percentiles when appropriate. Categorical data is presented as number (n) and percentages (%). The statistical difference between groups were tested with Pearson's chi-square (categorical data) and with Mann Whitney U-test (continuous variables). Fisher's exact test was used when appropriate. Two-tailed P-value < 0.05 was considered statistically significant.

## RESULTS

### Number of EMS missions and characteristics

A total of 74 576 EMS missions were screened for the study: 14 320 missions during the study period (1 March – 30 June 2020) and 60 256 in the control period (1 March – 30 June 2016–2019). From these missions, 49.9% of the patients were males with a mean age of 58.9 years (± 24.7).

In March and April 2020, the number of EMS missions per day decreased by 5.7% and 13.6%, respectively, compared to the control period. On 24 April 2020, the number of missions was 26% lower in the study period than in the control period [Figure 1]. The crude and age adjusted rates of EMS missions were significantly lower in the study period compared to the control period. The proportion of missions outside the office hours and non-urgent missions increased in the study period [Table 2].

### Time intervals

All time intervals (emergency call to dispatch, dispatch time, en-route time, time at scene and hospital handover) prolonged in the COVID-19 period [Table 2]. The highest increases in dispatch delays were seen in mission codes hypo-/hyperglycemia (+1:20min, p=0.002), dyspnea (+1:03, p<0.001), seizure (+0:23min, p=0.007), stroke (+0:13min p=0.001) and chest pain (+0:16min, p<0.001). There was no increase in the dispatch times in motor vehicle accidents (+0:21, p=0.08) or cardiac arrests (+0:06min, p=0.40).

### Distribution of mission codes

The changes in the distribution of mission codes are illustrated in **Table 3**. Numerically highest changes were the increases in dispatches to dyspnea (+0.7%, p=0.004), chest pain (+0.7%, p=0.005), stroke (+0.7%, p<0.001), traumas (+0.9%, p=0.003) and mental health (+1.2%, p<0.001), and decrease to interfacility transfers (-0.9%, p=0.001). It should be noted that when doing comparison between years 2020 and 2019 only, the differences in dyspnea (p=0.111), stroke (p=0.096) and traumas (p=0.233) were not statistically significant.

http://mc.manuscriptcentral.com/ejph

**Non-transported patients**

The proportion of non-transported patients (NTP) of the all EMS dispatches increased in the study period compared to the control period (39.9% vs 36.1%, p<0.001). The difference was also statistically significant when comparing the years 2020 and 2019 only (39.9% vs 36.9%, p<0.001). EMS consulted physicians more frequently during the study period regarding the non-transport decision (43.7% vs 41.4%, p=0.002), and the proportion of NTP not needing any medical intervention by EMS increased (42.2% vs 39.1%, p<0.001).

**COVID-19 patients encountered by EMS**

A total of 135 suspected COVID-19 cases (U07.2, ICD-10) and eleven confirmed (U07.1) cases were encountered by EMS during the study period. The median age of the confirmed COVID-19 cases was 47.3 [29.7–55.3] years and six (54.4%) of them were males. The most frequent transportation code was dyspnea (37.5 %), and three patients were released at scene without transportation. There were no differences between the NEWS scores (p=0.721) in patients with COVID-19 infection compared to the controls. Median dispatch time (7:52min, p=0.018) and hospital handover time (+11:51min, p<0.001) was prolonged with the confirmed COVID-19 patients.

**DISCUSSION**

In the present study, we found a substantial reduction in the number of EMS missions after the beginning of the COVID-19 pandemic and the declaration of the national state of emergency. The reduction lasted until the first wave of the pandemic faded and the social restrictions were lifted. The reduction can be explained by the decreased number of missions involving elderly patients. Moreover, all the measured mission time intervals prolonged significantly during the study period, also in the time-critical calls. Small changes were seen in the distribution of mission codes as well.

Our finding of the sudden drop in the number of attending patients is convergent with the studies from emergency departments [3, 4, 10], but results regarding the EMS are contradictory. Slavova et al. (2020) found a similar, more than 20% reduction in the number of EMS calls after the state of emergency had been declared in Kentucky, United States [11]. On the other hand, in Northern Italy where the pandemic struck viciously, a notable increase in the number of ambulance responds was discovered [7]. The pandemic situation was far worse in Italy than in Finland. In the present study, the number of missions decreased soon after the Finnish government declared a state of emergency, even though the national COVID-19 cases reached its peak approximately a month later, in early April. The state of emergency was lifted on 16 June 2020, and the differences in the number of missions in May and June are not statistically significant compared to the control period [Table 1]. These findings could be explained by the fact that the emergency laws and social restrictions have, itself, a major impact on the use of EMS, rather than only on the actual number of COVID-19 patients. Additionally, to our knowledge, this is the first paper to describe that the decrease in the number of patients is mainly composed by a drop in the rate of individuals aged over 65 years [Table 1]. Previously it is known that our study area presents with a high usage of EMS, especially in low-income areas and with elderly population [12]. The rate of missions was similar between people with 20 – 60 years of age.

One possible explanation to the reported decrease in the number of missions is that citizens have an elevated threshold to make an emergency call during a pandemic and a state of emergency. This raises a concern that there is a delay to seek help with time-critical medical conditions, such as stroke or acute myocardial infarction (AMI). Holmes et al. (2020) screened the number of EMS responses to strokes and AMIs and found no difference between the pre- and ongoing pandemic periods [13]. Correspondingly in the present study, there were no differences in the rate of dispatches to stroke or chest pain in the study periods [Table 3]. However, this aspect does not detect the possible delayed admissions from the onset of symptoms. In three European countries, a reduced number of stroke patients accessing the neurological emergency pathways was observed. This could be partly explained by the national campaigns' aim to reduce "unnecessary" ED visits during the pandemic, which may have caused delays in the admissions of stroke patients [14].

Guidelines for the rational use of personal protective equipment (PPE) during the COVID-19 pandemic has been published [15]. Following those guidelines and the Northern Ostrobothnia Hospital District protocol,

EMS provider used PPE in contact with patients suffering from any clinically suspected infection, and if the patient was older than 70 years regardless of the symptoms. The "coronavirus suspicion" given to EMS unit by EMCC in the presence of any infectious symptoms was adjusted to high sensitivity, producing a high number of false positives, for example, to patients with asthma or COPD. This policy of PPE usage may have been the leading cause of the prolonged time intervals, especially missions where the PPE for aerosol generating procedures had to be used. Nevertheless, our study did not aim to investigate if the prolonged response times had any impact on the patient outcome.

Regarding the increased hospital handover time, there may be two factors contributing: First, new and possibly impractical protocols for the patients with a suspected infection in the ED, and second, increased need for ambulance disinfection. These findings point out the importance of functional, evidence-based protocols for efficient and timesaving PPE use and disinfection of ambulances and equipment. However, the dispatch time to cardiac arrests was not prolonged, a promising finding despite the COVID-19 guidelines by the European Resuscitation Council for the use of PPE in out-of-hospital cardiac arrests [16].

There are preliminary findings of increased alcohol misuse during the COVID-19 lockdowns and social distancing [17, 18]. An observational study from the United States noted an increase in opioid overdose-calls soon after the implementation of social restrictions [11]. However, in the present study we found no increase in substance misuse related missions [Tables 3], but rather a decline in the number of intoxicated (≥ 0.5‰) patients encountered by EMS (15.0% vs 16.2%, p<0.001). This result may be explained by an increased threshold to make emergency calls, but because of the subtle differences between the study groups, caution must be applied when interpreting these results. Last, the proportion of mental health calls increased by 1.2 % during the study period [Table 3]. This finding further supports the hypothesis that there is an association between mental health problems and the COVID-19 pandemic [18], possibly due to the social restrictions and global fear of a novel, potentially dangerous infectious disease. Especially worrisome fact was the decline of median age among mental health patients (41.4 vs 38.0, p=0.017) during pandemic. Overall, the presented data suggest that in addition to the patients with the actual infection, the challenge among the next COVID-19 waves and other pandemics may also be the various deleterious effects of the social restrictions and lockdowns.

**Limitations**

The main limitation of the present study covers the selection of the control group. The control group was composed from missions between the years 2016 – 2019 during corresponding months as in the study period. The main benefit of this approach is to reduce short-term randomness in the measurable statistics and control the seasonal fluctuation. However, the total number of EMS missions has been growing steadily during the past years [19]. Similarly, there may be some factors affecting the change in dispatch codes and patient characteristics, such as the increasing median age of EMS patients. Therefore, we were not able to reliably eliminate these possible confounding factors, and there may be several other factors affecting the observed findings than the COVID-19 pandemic. Despite this bias, the main finding concerning the reduced number of EMS missions during the COVID-19 pandemic remains. If we extrapolate the yearly growth seen in the number of missions in the original data, the decrease is even higher (13% – 18%) [Supplementary Figure 1].

**Conclusions**

Our study shows a substantial decrease in the number of EMS missions and prolonged time intervals in the study area after the beginning of the COVID-19 pandemic and the declaration of a state of emergency. EMS encountered only a few patients with confirmed COVID-19 infection. These results suggest that the COVID-19 pandemic and social restrictions may play a significant effect on the need for EMS. There is an indispensable need of further development of EMS protocols and strategies for preparation to oncoming waves of the COVID-19 and other novel pandemics.

**Funding**

This study received no specific grant from any funding agency in the public, commercial, or not-for-profit sectors.

**Conflicts of interests**

The authors declare no conflict of interest.

**Contribution** LL, JL and LR designed the study. AE collected the data from electronic EMS journal and LL performed the data analysis. LL and SL wrote the initial draft of the manuscript and LR, JL and TK contributed to redrafting and refinement of the manuscript. All authors read and approved the final manuscript.

**Acknowledgment**

The authors wish to thank SS, Research Nurse, for her kind assistance in the data collection.

**Data availability statement**

Data are available on reasonable request from the corresponding author and with approval from the administration of OUH.

**KEY POINTS**

- **The number of EMS missions decreased after the beginning of the COVID-19 pandemic.**
- **The decline was mainly produced by a decrease in the rate of elderly patients.**
- **Social restrictions and lockdowns are probably responsible for some of the changes in healthcare during COVID-19.**
- **When implementing national social restrictions and healthcare protocols during pandemic, attention is needed to evaluate how these affect critically ill patients.**

## References

1. World Health Organization. Rolling updates on coronavirus disease (COVID-19). Accessed 6.10.2020. Available: https://www.who.int/emergencies/diseases/novel-coronavirus-2019/events-as-they-happen

2. Li H, Liu S, Yu X, et al. Coronavirus disease 2019 (COVID-19): current status and future perspectives. *Int J Antimicrob Agents* 2020;55(5):105951.

3. Thornton J. Covid-19: A&E visits in England fall by 25% in week after lockdown. *BMJ* 2020;6;369:m1401.

4. Cao Y, Li Q, Chen J, et al. Hospital Emergency Management Plan During the COVID-19 Epidemic. Academic emergency medicine: *official journal of the Society for Academic Emergency Medicine* 2020;27(4):309-311.

5. Thu TPB, Ngoc PNH, Hai NM, et al. Effect of the social distancing measures on the spread of COVID-19 in 10 highly infected countries. *Sci Total Environ* 2020;10;742:140430.

6. Dami F and Berthoz V. Lausanne medical dispatch centre's response to COVID-19. *Scand J Trauma Resusc Emerg Med* 2020;13;28(1):37.

7. Perlini S, Canevari F, Cortesi S, et al. Emergency Department and Out-of-Hospital Emergency System (112-AREU 118) integrated response to Coronavirus Disease 2019 in a Northern Italy centre. *Intern Emerg Med* 2020;15(5):825-833.

8. Finnish Institute for Health and Welfare (THL). COVID-19 cases in the infectious diseases registry. Accessed 27.6.2020. Available: https://sampo.thl.fi/pivot/prod/fi/epirapo/covid19case/fact_epirapo_covid19case

9. Pekanoja S, Hoikka M, Kyngas H, et al. Non-transport emergency medical service missions – a retrospective study based on medical charts. *Acta Anaesthesiol Scand* 2018;62(5):701-708.

10. Vanni G, Legramante JM, Pellicciaro M, et al. Effect of Lockdown in Surgical Emergency Accesses: Experience of a COVID-19 Hospital. *In Vivo* 2020;34(5):3033-3038.

11. Slavova S, Rock P, Bush HM, et al. Signal of increased opioid overdose during COVID-19 from emergency medical services data. *Drug Alcohol Depend* 2020;214:108176.

12. Aitavaara-Anttila M, Liisanantti J, Ehrola A, et al. Use of prehospital emergency medical services according to income of residential area. *Emerg Med J* 2020;37(7):429-433.

13. Holmes JL, Brake S, Docherty M, et al. Emergency ambulance services for heart attack and stroke during UK's COVID-19 lockdown. *Lancet* 2020;395(10237):e93-e94.

14. Bersano A, Kraemer M, Touzé E, et al. Stroke care during the COVID-19 pandemic: experience from three large European countries*. European journal of neurology* 2020;10.1111/ene.14375.

15. Mahmood SU, Crimbly F, Khan S, et al. Strategies for Rational Use of Personal Protective Equipment (PPE) Among Healthcare Providers During the COVID-19 Crisis. *Cureus* 2020;12(5):e8248.

16. European Resuscitation Council. COVID-19 Guidelines. Risks associated with cardiopulmonary resuscitation (CPR) in patients with COVID-19. *ERC* 2020.

17. The Lancet Gastroenterology & Hepatology. Drinking alone: COVID-19, lockdown, and alcohol-related harm. Editorial. *The Lancet Gastroenterology & Hepatology* 2020;5(7):625.

18. Czeisler MÉ, Lane RI, Petrosky E, et al. Mental Health, Substance Use, and Suicidal Ideation During the COVID-19 Pandemic - United States, June 24-30, 2020. *MMWR Morb Mortal Wkly Rep* 2020;69(32):1049-1057.

19. Andrew E, Nehme Z, Cameron P, et al. Drivers of Increasing Emergency Ambulance Demand. *Prehosp Emerg Care* 2020;24(3):385.

**Table 1. Number of EMS missions per day**

| | Mean number of EMS missions per day<br>n (± SD) | | |
|---|---|---|---|
| | Control period<br>1 March – 30 June<br>2016/17/18/19<br>(n=60 256) | Study period<br>1 March – 30 June 2020<br>(n=14 320) | p-value |
| March | 125.2 (± 13.7) | 118.1 (± 17.4) | 0.011 |
| April | 123.9 (± 15.4) | 107.1 (± 13.7) | < 0.001 |
| May | 119.3 (± 13.4) | 115.8 (± 13.4) | 0.251 |
| June | 125.3 (± 17.3) | 128.6 (± 16.1) | 0.174 |
| | | | |
| | Rate of EMS missions calculated for 1000 persons per year<br>n (95% CI) | | |
| Crude rate | 144.8 (144.3 – 145.2) | 137.8 (137.0 – 138.7) | < 0.001 |
| Age adjusted rate | 308.0 (307.1 – 309.0) | 288.4 (286.6 – 290.2) | < 0.001 |
| Rate for population > 65 years | 390.0 (385.5 – 394.4) | 360.7 (352.2 – 369.2) | < 0.001 |
| Rate for population 20 – 65 years | 117.3 (116 – 118.7) | 114.3 (111.7 – 117.0) | 0.053 |
| Rate for population < 20 years | 49.0 (47.7 – 50.3) | 36.7 (36.7 – 41.5) | 0.009 |

**Table 2. Characteristics of EMS missions**

| | Control period 1 March – 30 June 2016/17/18/19 (n=60 256) | COVID-19 period 1 March – 30 June 2020 (n=14 320) | Odds Ratio (95% confidence interval) | p-value | Missing |
|---|---|---|---|---|---|
| **Patient characteristics** [a] | | | | | |
| Age (± SD) | 58.9 (± 24.7) | 59.1 (± 24.4) | N/A | 0.921 | 270 |
| Gender (male) | 29 051 (49.7 %) | 7093 (50.8 %) | 1.05 (1.02–1.09) | < 0.001 | 440 |
| | | | | | |
| **Time of the dispatch** | | | | | 32 |
| 8am – 16pm | 28 519 (47.4 %) | 6537 (45.7 %) | 0.93 (0.90–0.97) | < 0.001 | |
| 16pm – 22pm | 16 946 (28.1 %) | 4167 (29.1 %) | 1.05 (1.01–1.09) | 0.020 | |
| 22pm – 08am | 14 760 (24.5 %) | 3615 (25.2 %) | 1.04 (0.99–1.09) | 0.062 | |
| | | | | | |
| **Priority** [b] | | | | | 25 |
| A (urgent, suspected disturbance of vital signs) | 743 (4.7 %) | 503 (3.5 %) | 0.74 (0.66–0.83) | < 0.001 | |
| B (urgent) | 5110 (32.4 %) | 4250 (29.7 %) | 0.88 (0.84–0.93) | < 0.001 | |
| C (response under 30 minutes) | 4995 (31.7 %) | 4744 (33.1 %) | 1.07 (1.02–1.12) | 0.006 | |
| D (response under 2 hours) | 4928 (31.2 %) | 4822 (33.7 %) | 1.12 (1.07–1.17) | < 0.001 | |
| | | | | | |
| **Time intervals as minutes (medians)** [c] | | | | | |
| Emergency call to dispatch | 3:01 [2:08 – 4:24] | 3:40 [2:10 – 5:48] | N/A | < 0.001 | |
| Dispatch time | 2:35 [1:19 – 4:26] | 3:09 [1:37 – 5:22] | N/A | < 0.001 | 2581 |
| Time en-route | 7:25 [4:42 – 11:33] | 7:40 [4:54 – 12:19] | N/A | < 0.001 | 5456 |
| Time at scene | 23:31 [16:10 – 32:12] | 25:44 [17:35 – 36:00] | N/A | < 0.001 | 10 050 |
| Handover time at hospital | 15:49 [11:39 – 20:52] | 19:40 [14:50 – 25:35] | N/A | < 0.001 | 16 347 |
| | | | | | |
| **Others** | | | | | |
| Physician consulted | 14 639 (24.3 %) | 3828 (26.7 %) | 1.14 (1.09–1.19) | < 0.001 | |
| Physician at scene | 373 (0.6 %) | 53 (0.4 %) | 0.60 (0.45–0.80) | < 0.001 | |

[a] Missions without a patient excluded

[b] Missions before 1 January 2019 excluded due to the change in the EMCC dispatch system

[c] "Emergency call to dispatch": time between the beginning of the initial emergency call and transmitting the mission to EMS unit; "Dispatch time": time between EMS unit alarmed by EMCC and unit en-route; "Time at scene": time between the arrival of the EMS unit at the scene and the beginning of transportation or unit released from the scene without transportation; "Handover time at hospital"; time between the arrival to the hospital and the EMS unit leaving the hospital capable to receive the next EMS mission.

**Table 3. Dispatch codes**

| | Control period 1 March – 30 June 2016/17/18/19 (n=60 256) | COVID-19 period 1 March – 30 June 2020 (n=14 320) | Odds Ratio (95% confidence interval) | p-value |
|---|---|---|---|---|
| | | | | |
| **Organ specific calls** | | | | |
| Cardiac arrest or ongoing CPR | 292 (0.5%) | 86 (0.6%) | 1.24 (0.98–1.58) | 0.086 |
| Unconsciousness | 387 (0.6%) | 59 (0.4%) | 0.64 (0.49–0.84) | 0.001 |
| Dyspnea | 4032 (6.7%) | 1056 (7.4%) | 1.11 (1.04–1.19) | 0.004 |
| Chest pain | 4763 (7.9%) | 1234 (8.6%) | 1.10 (1.03–1.17) | 0.005 |
| Stroke | 2020 (3.4%) | 588 (4.1%) | 1.23 (1.12–1.36) | < 0.001 |
| Abdominal pain | 2087 (3.5%) | 525 (3.7%) | 1.06 (0.96–1.17) | 0.234 |
| Headache | 914 (1.5%) | 177 (1.2%) | 0.81 (0.69–0.96) | 0.013 |
| Pregnancy or birth | 364 (0.6%) | 101 (0.7%) | 1.17 (0.94–1.46) | 0.173 |
| | | | | |
| **Trauma** | | | | |
| Airway obstruction (foreign object, drowning, hanging) | 236 (0.4%) | 47 (0.3%) | 0.84(0.61–1.15) | 0.290 |
| Other traumas | 414 (0.7%) | 112 (0.8%) | 1.14 (0.92–1.41) | 0.221 |
| Trauma related calls (falls, wounds, non-violent traumas) | 7834 (13.0%) | 1997 (13.9%) | 1.08 (1.03–1.14) | 0.003 |
| Motor vehicle accident | 2215 (3.7%) | 477 (3.3%) | 0.90 (0.82–1.00) | 0.470 |
| House fire | 164 (0.3%) | 44 (0.3%) | 1.13 (0.81–1.58) | |
| Violent traumas (gunshot, stabbing, assault) | 997 (1.7%) | 200 (1.4%) | 0.84 (0.72–0.98) | 0.028 |
| | | | | |
| **Non-organ specific calls and nonspecific symptoms** | | | | |
| Musculoskeletal pain | 3565 (5.9%) | 834 (5.8%) | 0.98 (0.91–1.06) | 0.692 |
| Substance abuse, overdose | 2039 (3.4%) | 444 (3.1%) | 0.91 (0.82–1.01) | 0.093 |
| Bleeding (mouth, vaginal/rectal, nose) | 1279 (2.1%) | 358 (2.5%) | 1.18 (1.05–1.33) | 0.006 |
| Undefined illness (sudden / chronic) | 13 518 (22.4%) | 3131 (21.9%) | 0.97 (0.93–1.01) | 0.144 |
| Epileptic seizure | 1474 (2.4%) | 306 (2.1%) | 0.87 (0.77–0.99) | 0.031 |
| Hypo- or hyperglycemia | 640 (1.1%) | 107 (0.7%) | 0.70 (0.57–0.86) | 0.001 |
| Allergic reaction | 456 (0.8%) | 82 (0.6%) | 0.76 (0.60–0.96) | 0.020 |
| Vomiting or diarrhea | 1726 (2.9%) | 360 (2.5%) | 0.87 (0.78–0.98) | 0.023 |
| Mental disorder | 2668 (4.4%) | 799 (5.6%) | 1.28 (1.18–1.38) | < 0.001 |
| Interfacility transfer | 5402 (9.0%) | 1155 (8.1%) | 0.89 (0.83–0.95) | 0.001 |
| | | | | |
| Other calls | 744 (1.2%) | 40 (0.3%) | 0.22 (0.16–0.31) | < 0.001 |
| Missing data | 26 | 1 | | |

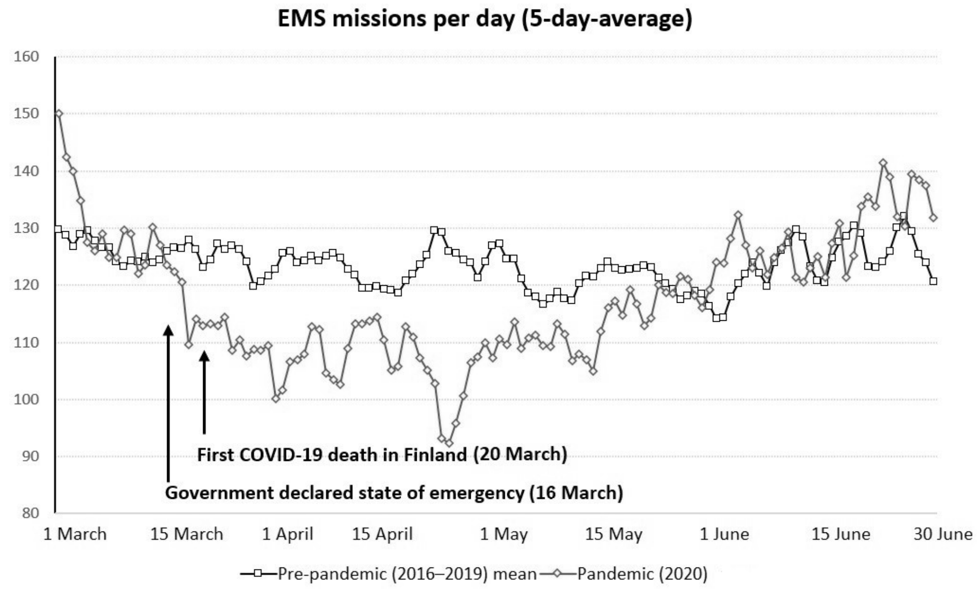

Figure 1. Change in the number of EMS missions per day between pre-pandemic and pandemic period.

279x170mm (96 x 96 DPI)
