# Early impact of the COVID-19 pandemic and social restrictions on ambulance missions

**DOI:** 10.1093/eurpub/ckab065

**Published:** 2021-04-15

**Authors:** Lauri Laukkanen, Sanna Lahtinen, Janne Liisanantti, Timo Kaakinen, Ari Ehrola, Lasse Raatiniemi

**Affiliations:** 1 Research Group of Surgery, Anaesthesiology and Intensive Care Medicine, Medical Research Center of Oulu University, Oulu University Hospital, Oulu, Finland; 2 Department of Anaesthesiology, Oulu University Hospital, Oulu, Finland; 3 Emergency Medical Services, Oulu-Koillismaa Rescue Department, Oulu University Hospital, Oulu, Finland; 4 Centre for Pre-Hospital Emergency Care, Oulu University Hospital, Oulu, Finland

## Abstract

**Background:**

The SARS-CoV-2 coronavirus disease 2019 (COVID-19) has had a major impact on health care services globally. Recent studies report that emergency departments have experienced a significant decline in the number of admitted patients in the early phase of the pandemic. To date, research regarding the influence of COVID-19 on emergency medical services (EMS) is limited. This study investigates a change in the number and characteristics of EMS missions in the early phase of the pandemic.

**Methods:**

All EMS missions in the Northern Ostrobothnia region, Finland (population 295 500) between 1 March to 30 June 2020 were screened and analyzed as the study group. A control group was composed from the EMS calls between the corresponding months in the years 2016–19.

**Results:**

A total of 74 576 EMS missions were screened for the study. Within the first 2 months after the first COVID-19 cases in the study area, the decline in the number of EMS missions was 5.7–13% compared with the control group average. EMS time intervals (emergency call to dispatch, dispatch, en-route, on-scene and hospital handover) prolonged in the COVID-19 period. Dispatches concerning mental health problems increased most in the study period (+1.2%, *P* < 0.001). Only eleven confirmed COVID-19 infections were encountered by EMS in the study period.

**Conclusion:**

Our findings suggest that the present COVID-19 pandemic and social restrictions lead to changes in the EMS usage. These preliminary findings emphasize the importance of developing new strategies and protocols in response to the oncoming pandemic waves.

## Introduction

In December 2019, a novel coronavirus (SARS-CoV-2) was identified in Wuhan, China, and it evoked a global pandemic in the spring of 2020.^1^ The coronavirus disease 2019 (COVID-19) is characterized by asymptomatic infection to severe pneumonia and respiratory failure.^2^ To date (13 March 2021), over 118 million cases and over 2 million fatalities worldwide have been reported so far.^1^

A decline in the number of patients presenting to emergency department (ED) has been reported in the early phase of the COVID-19 pandemic.^3^^,^^4^ Several efforts in the forms of social restrictions and nationwide lockdowns have been implemented trying to reduce the spread of this disease.^5^ In the context of emergency medical services (EMS), there is a limited amount of research concerning the effect of COVID-19. A single study from Switzerland reported an increase in the number of emergency calls and ambulance dispatches in the early weeks of the pandemic.^6^ Although, contrary findings have also been reported.^7^

The objective of this study was to examine if there was a change in the number and characteristics of EMS missions and patients in early phase of the COVID-19 pandemic compared with previous years. Further, our aim was to investigate the prevalence of suspected and confirmed COVID-19 cases in EMS patients.

## Methods

### Study design and ethics

The study design is an observational, retrospective study. The approval was obtained from the administration of Oulu University Hospital (OUH; Reference number 54/2019, updated on 17 June 2020). According to the local policies and due to the retrospective study design, approval from the ethics committee and consent from the patients were not needed. The STROBE checklist for observational studies was used in reporting this study.

### Study area and COVID-19 spread

The study was carried out in the Northern Ostrobothnia region, in Northern Finland which is a mixed rural–urban region with 295 500 inhabitants and population density of 12.8 km^2^. The first COVID-19 case in the study area occurred on 6 March 2020, followed by a total of 136 confirmed cases during the study period. Correspondingly, 7271 COVID-19 cases occurred in Finland during the same period.^8^ In early March 2020, the number of COVID-19 cases increased rapidly in Finland, and health care services could not respond to the rising demand of COVID testing. The Finnish Institute for Health and Welfare (THL) calculated models for the expected progression of the pandemic, and the risk of an overcapacity of ICU’s and hospital network was considered as a substantial threat. In response, on 16 March 2020, the Finnish Government declared a state of emergency, forcing various restrictions on Finnish society, including education, public and cultural services, public gatherings and travel (Supplementary table S1). The state of emergency was lifted on 16 July 2020.

### EMS and hospital network

All emergency calls (EMS, police and fire) in the study area are responded by the emergency medical communication centre (EMCC), where the call is evaluated by an emergency dispatcher. Dispatcher determines mission code and priority and dispatches the corresponding organization. Four classes of priority are used, urgent priorities A (highest) and B, and non-urgent priorities C and D (lowest).

A tiered EMS system includes basic- and advanced life support (BLS and ALS) ambulances, field supervisor, and physician-staffed helicopter EMS. BLS units are operated by emergency medical technicians or firefighters, and ALS includes at least one paramedic. The local non-transportation guidelines have an option that the EMS crew may refer patients to hospital by other means than ambulance or treat them at the scene. The non-transport rate in the study area is generally around 38%.^9^ There is a tertiary-level care hospital (OUH) and several smaller municipal health centres providing primary health care services in the study area. During the COVID-19 outbreak, ED in OUH was divided into areas for infectious and non-infectious patients, and few of the smaller health centres in the study area were focused to treat only infectious patients. In EMCC, routinely asked questions about infectious symptoms and COVID-19 exposure were added to the EMCC key-question protocol of EMS calls. In presence of these symptoms or exposure, the dispatcher gave a ‘coronavirus suspicion’ to the responding EMS unit.

### Setting and participants

All EMS missions in the study area between 1 March and 30 June 2020 were retrospectively screened and were assigned to the study group. According to the THL statistics, the first wave of COVID-19 took place during this period, a peak of cases occurring in early April.^8^ The control group for comparison comprised EMS missions in the same area between the corresponding months (1 March to 30 June) in the years 2016–19.

### Data collection and variables

The EMS provider in the study area uses an electronic patient documenting system (Merlot Medi, CGI© Suomi Oy). The Hospital District of Northern Ostrobothnia has an electronic patient management system (Oberon, Uranus) for administrative registration of hospital treatment, diagnosis, and care pathways. Data concerning the number of missions per day, timeline of the dispatch, mission code, priority, characteristics and clinical information were retrieved from the EMS database. Information of the suspected or confirmed COVID-19 infection were collected from the hospital district database.

### Statistical analysis

The statistical analysis was performed using the SPSS statistics software (IBM^©^ Corp., SPSS Statistics for Windows, version 25.0). For the analysis of the change in the number of EMS missions, a mean number of daily EMS missions were calculated for each month. For further analysis, missions within the years 2016–19 were summed to a single control group. The population data including the age groups were retrieved from the open database of statistics Finland. The rates of EMS missions for 1000 inhabitants per year were calculated using the population of the area in the year 2018 as the index year.

Continuous data are presented as means and ±SD or medians and 25 and 75 percentiles when appropriate. Categorical data are presented as number (*n*) and percentages (%). The statistical difference between groups was tested with Pearson’s chi-square (categorical data) and with Mann–Whitney U-test (continuous variables). Fisher’s exact test was used when appropriate. Two-tailed *P* values < 0.05 was considered statistically significant.

## Results

### Number of EMS missions and characteristics

A total of 74 576 EMS missions were screened for the study: 14 320 missions during the study period (1 March to 30 June 2020) and 60 256 in the control period (1 March to 30 June 2016–19). From these missions, 49.9% of the patients were males with a mean age of 58.9 years (± 24.7).

**Figure 1:**
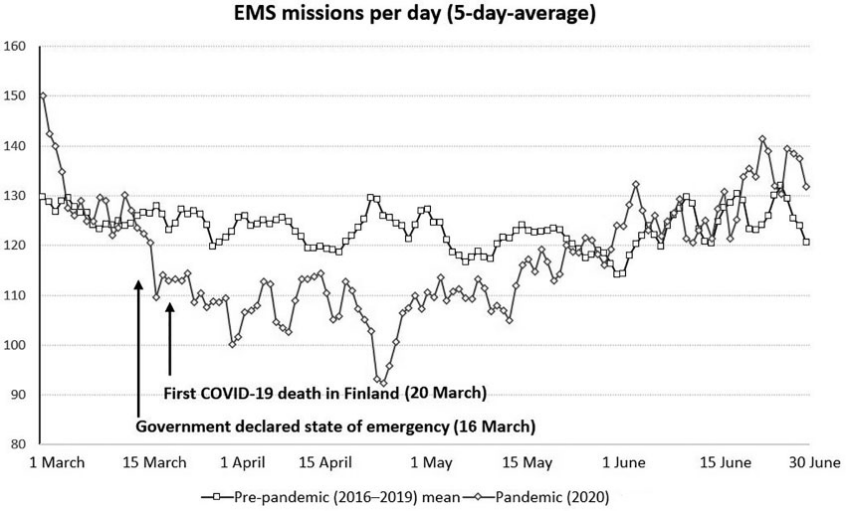
Change in the number of EMS missions per day between pre-pandemic and pandemic period

In March and April 2020, the number of EMS missions per day decreased by 5.7% and 13.6%, respectively, compared with the control period. On 24 April 2020, the number of missions was 26% lower in the study period than in the control period (figure 1). The crude and age adjusted rates of EMS missions were significantly lower in the study period compared with the control period. The proportion of missions outside the office hours and non-urgent missions increased in the study period (table 1).

**Table 1:** Characteristics of EMS missions

	Control period	COVID-19 period	Odds ratio (95% CI)	*p*-value	Missing
	1 March to 30 June 2016–19 (*n* = 60 256)	1 March to 30 June 2020 (*n* = 14 320)			
Patient characteristics^a^					
Age (± SD)	58.9 (± 24.7)	59.1 (± 24.4)	N/A	0.921	270
Gender (male)	29 051 (49.7 %)	7093 (50.8 %)	1.05 (1.02–1.09)	<0.001	440
Time of the dispatch					32
8 am—16 pm	28 519 (47.4 %)	6537 (45.7 %)	0.93 (0.90–0.97)	<0.001	
16 pm—22 pm	16 946 (28.1 %)	4167 (29.1 %)	1.05 (1.01–1.09)	0.020	
22 pm—08 am	14 760 (24.5 %)	3615 (25.2 %)	1.04 (0.99–1.09)	0.062	
Priority^b^					25
A (urgent, suspected disturbance of vital signs)	743 (4.7 %)	503 (3.5 %)	0.74 (0.66–0.83)	<0.001	
B (urgent)	5110 (32.4 %)	4250 (29.7 %)	0.88 (0.84–0.93)	<0.001	
C (response under 30 min)	4995 (31.7 %)	4744 (33.1 %)	1.07 (1.02–1.12)	0.006	
D (response under 2 h)	4928 (31.2 %)	4822 (33.7 %)	1.12 (1.07–1.17)	<0.001	
Time intervals as minutes (medians)^c^					
Emergency call to dispatch	3:01 [2:08–4:24]	3:40 [2:10–5:48]	N/A	<0.001	
Dispatch time	2:35 [1:19–4:26]	3:09 [1:37–5:22]	N/A	<0.001	2581
Time en-route	7:25 [4:42–11:33]	7:40 [4:54–12:19]	N/A	<0.001	5456
Time at scene	23:31 [16:10–32:12]	25:44 [17:35–36:00]	N/A	<0.001	10 050
Handover time at hospital	15:49 [11:39–20:52]	19:40 [14:50–25:35]	N/A	<0.001	16 347
Others					
Physician consulted	14 639 (24.3 %)	3828 (26.7 %)	1.14 (1.09–1.19)	<0.001	
Physician at scene	373 (0.6 %)	53 (0.4 %)	0.60 (0.45–0.80)	<0.001	

a Missions without a patient excluded.

b Missions before 1 January 2019 excluded due to the change in the EMCC dispatch system.

c ’Emergency call to dispatch’: time between the beginning of the initial emergency call and transmitting the mission to EMS unit; ‘Dispatch time’: time between EMS unit alarmed by EMCC and unit en-route; ‘Time at scene’: time between the arrival of the EMS unit at the scene and the beginning of transportation or unit released from the scene without transportation; ‘Handover time at hospital’; time between the arrival to the hospital and the EMS unit leaving the hospital capable to receive the next EMS mission.

### Time intervals

All time intervals (emergency call to dispatch, dispatch time, en-route time, time at scene and hospital handover) prolonged in the COVID-19 period (table 1). The highest increases in dispatch delays were seen in mission codes hypo-/hyperglycaemia (+1:20 min, *P* = 0.002), dyspnoea (+1:03 min, *P* < 0.001), seizure (+0:23 min, *P* = 0.007), stroke (+0:13 min *P* = 0.001) and chest pain (+0:16 min, *P* < 0.001). There was no increase in the dispatch times in motor vehicle accidents (+0:21 min, *P* = 0.08) or cardiac arrests (+0:06min, *P* = 0.40).

### Distribution of mission codes

The changes in the distribution of mission codes are illustrated in table 2. Numerically highest changes were the increases in dispatches to dyspnoea (+0.7%, *P* = 0.004), chest pain (+0.7%, *P* = 0.005), stroke (+0.7%, *P* < 0.001), traumas (+0.9%, *P* = 0.003) and mental health (+1.2%, *P* < 0.001), and decrease to interfacility transfers (−0.9%, *P* = 0.001). It should be noted that when doing comparison between years 2020 and 2019 only, the differences in dyspnoea (*P* = 0.111), stroke (*P* = 0.096) and traumas (*P* = 0.233) were not statistically significant.

**Table 2:** Dispatch codes

	Control period	COVID-19 period	Odds ratio (95% CI)	*P*-value
**1 March to 30 June 2016–19 (*n* = 60 256)**	**1 March to 30 June 2020 (*n* = 14 320)**			
Organ-specific calls				
Cardiac arrest or ongoing cardiopulmonary resuscitation	292 (0.5%)	86 (0.6%)	1.24 (0.98–1.58)	0.086
Unconsciousness	387 (0.6%)	59 (0.4%)	0.64 (0.49–0.84)	0.001
Dyspnoea	4032 (6.7%)	1056 (7.4%)	1.11 (1.04–1.19)	0.004
Chest pain	4763 (7.9%)	1234 (8.6%)	1.10 (1.03–1.17)	0.005
Stroke	2020 (3.4%)	588 (4.1%)	1.23 (1.12–1.36)	< 0.001
Abdominal pain	2087 (3.5%)	525 (3.7%)	1.06 (0.96–1.17)	0.234
Headache	914 (1.5%)	177 (1.2%)	0.81 (0.69–0.96)	0.013
Pregnancy or birth	364 (0.6%)	101 (0.7%)	1.17 (0.94–1.46)	0.173
Trauma				
Airway obstruction (foreign object, drowning and hanging)	236 (0.4%)	47 (0.3%)	0.84(0.61–1.15)	0.290
Other traumas	414 (0.7%)	112 (0.8%)	1.14 (0.92–1.41)	0.221
Trauma-related calls (falls, wounds and non-violent traumas)	7834 (13.0%)	1997 (13.9%)	1.08 (1.03–1.14)	0.003
Motor vehicle accident	2215 (3.7%)	477 (3.3%)	0.90 (0.82–1.00)	0.470
House fire	164 (0.3%)	44 (0.3%)	1.13 (0.81–1.58)	
Violent traumas (gunshot, stabbing and assault)	997 (1.7%)	200 (1.4%)	0.84 (0.72–0.98)	0.028
Non-organ-specific calls and non-specific symptoms				
Musculoskeletal pain	3565 (5.9%)	834 (5.8%)	0.98 (0.91–1.06)	0.692
Substance abuse, overdose	2039 (3.4%)	444 (3.1%)	0.91 (0.82–1.01)	0.093
Bleeding (mouth, vaginal/rectal and nose)	1279 (2.1%)	358 (2.5%)	1.18 (1.05–1.33)	0.006
Undefined illness (sudden/chronic)	13 518 (22.4%)	3131 (21.9%)	0.97 (0.93–1.01)	0.144
Epileptic seizure	1474 (2.4%)	306 (2.1%)	0.87 (0.77–0.99)	0.031
Hypo- or hyperglycaemia	640 (1.1%)	107 (0.7%)	0.70 (0.57–0.86)	0.001
Allergic reaction	456 (0.8%)	82 (0.6%)	0.76 (0.60–0.96)	0.020
Vomiting or diarrhoea	1726 (2.9%)	360 (2.5%)	0.87 (0.78–0.98)	0.023
Mental disorder	2668 (4.4%)	799 (5.6%)	1.28 (1.18–1.38)	<0.001
Interfacility transfer	5402 (9.0%)	1155 (8.1%)	0.89 (0.83–0.95)	0.001
Other calls	744 (1.2%)	40 (0.3%)	0.22 (0.16–0.31)	<0.001
Missing data	26	1		

### Non-transported patients

The proportion of non-transported patients (NTPs) of the all EMS dispatches increased in the study period compared with the control period (39.9% vs. 36.1%, *P* < 0.001). The difference was also statistically significant when comparing the years 2020 and 2019 only (39.9% vs. 36.9%, *P* < 0.001). EMS consulted physicians more frequently during the study period regarding the non-transport decision (43.7% vs. 41.4%, *P* = 0.002), and the proportion of NTP not needing any medical intervention by EMS increased (42.2% vs. 39.1%, *P* < 0.001).

### COVID-19 patients encountered by EMS

A total of 135 suspected COVID-19 cases (U07.2, ICD-10) and 11 confirmed (U07.1) cases were encountered by EMS during the study period. The median age of the confirmed COVID-19 cases was 47.3 (29.7–55.3) years and 7 (54.4%) of them were males. The most frequent transportation code was dyspnoea (37.5%), and three patients were released at scene without transportation. There were no differences between the NEWS scores (*P* = 0.721) in patients with COVID-19 infection compared with the controls. Median dispatch time (7:52 min, *P* = 0.018) and hospital handover time (+11:51 min, *P* < 0.001) was prolonged with the confirmed COVID-19 patients.

## Discussion

In this study, we found a substantial reduction in the number of EMS missions after the beginning of the COVID-19 pandemic and the declaration of the national state of emergency. The reduction lasted until the first wave of the pandemic faded and the social restrictions were lifted. The reduction can be explained by the decreased number of missions involving elderly patients. Moreover, all the measured mission time intervals prolonged significantly during the study period, also in the time-critical calls. Small changes were seen in the distribution of mission codes as well.

Our finding of the sudden drop in the number of attending patients is convergent with the studies from EDs,^3^^,^^4^^,^^10^ but results regarding the EMS are contradictory. Slavova et al.^11^ found a similar, more than 20% reduction in the number of EMS calls after the state of emergency had been declared in Kentucky, USA. On the other hand, in Northern Italy where the pandemic struck viciously, a notable increase in the number of ambulance responds was discovered.^7^ The pandemic situation was far worse in Italy than in Finland. In this study, the number of missions decreased soon after the Finnish government declared a state of emergency, even though the national COVID-19 cases reached its peak approximately a month later, in early April. The state of emergency was lifted on 16 June 2020, and the differences in the number of missions in May and June are not statistically significant compared with the control period (table 3). These findings could be explained by the fact that the emergency laws and social restrictions have, itself, a major impact on the use of EMS, rather than only on the actual number of COVID-19 patients. Additionally, to our knowledge, this is the first paper to describe that the decrease in the number of patients is mainly composed by a drop in the rate of individuals aged over 65 years (table 3). Previously it is known that our study area presents with a high usage of EMS, especially in low-income areas and with elderly population.^12^ The rate of missions was similar between people with 20–60 years of age.

**Table 3:** Number of EMS missions per day

	Mean number of EMS missions per day *n* (± SD)		
	Control period	Study period	*P*-value
	1 March—30 June 2016–19 (*n* = 60 256)	1 March—30 June 2020 (*n* = 14 320)	
March	125.2 (± 13.7)	118.1 (± 17.4)	0.011
April	123.9 (± 15.4)	107.1 (± 13.7)	<0.001
May	119.3 (± 13.4)	115.8 (± 13.4)	0.251
June	125.3 (± 17.3)	128.6 (± 16.1)	0.174

	**Rate of EMS missions calculated for 1000 persons per year *n* (95% CI)**		

Crude rate	144.8 (144.3–145.2)	137.8 (137.0–138.7)	<0.001
Age adjusted rate	308.0 (307.1–309.0)	288.4 (286.6–290.2)	<0.001
Rate for population > 65 years	390.0 (385.5–394.4)	360.7 (352.2–369.2)	<0.001
Rate for population 20–65 years	117.3 (116–118.7)	114.3 (111.7–117.0)	0.053
Rate for population <20 years	49.0 (47.7–50.3)	36.7 (36.7–41.5)	0.009

One possible explanation to the reported decrease in the number of missions is that citizens have an elevated threshold to make an emergency call during a pandemic and a state of emergency. This raises a concern that there is a delay to seek help with time-critical medical conditions, such as stroke or acute myocardial infarction (AMI). Holmes et al.^13^ screened the number of EMS responses to strokes and AMIs and found no difference between the pre- and ongoing pandemic periods. Correspondingly in this study, there were no differences in the rate of dispatches to stroke or chest pain in the study periods (table 2). However, this aspect does not detect the possible delayed admissions from the onset of symptoms. In three European countries, a reduced number of stroke patients accessing the neurological emergency pathways were observed. This could be partly explained by the national campaigns’ aim to reduce ‘unnecessary’ ED visits during the pandemic, which may have caused delays in the admissions of stroke patients.^14^

Guidelines for the rational use of personal protective equipment (PPE) during the COVID-19 pandemic has been published.^15^ Following those guidelines and the Northern Ostrobothnia Hospital District protocol, EMS provider used PPE in contact with patients suffering from any clinically suspected infection, and if the patient was older than 70 years regardless of the symptoms. The ‘coronavirus suspicion’ given to EMS unit by EMCC in the presence of any infectious symptoms was adjusted to high sensitivity, producing a high number of false positives, e.g. to patients with asthma or chronic obstructive pulmonary disease (COPD). This policy of PPE usage may have been the leading cause of the prolonged time intervals, especially missions where the PPE for aerosol generating procedures had to be used. Nevertheless, our study did not aim to investigate if the prolonged response times had any impact on the patient outcome.

Regarding the increased hospital handover time, there may be two factors contributing: First, new and possibly impractical protocols for the patients with a suspected infection in the ED, and second, increased need for ambulance disinfection. These findings point out the importance of functional, evidence-based protocols for efficient and timesaving PPE use and disinfection of ambulances and equipment. However, the dispatch time to cardiac arrests was not prolonged, a promising finding despite the COVID-19 guidelines by the European Resuscitation Council for the use of PPE in out-of-hospital cardiac arrests.^16^

There are preliminary findings of increased alcohol misuse during the COVID-19 lockdowns and social distancing.^17^^,^^18^ An observational study from the USA noted an increase in opioid overdose-calls soon after the implementation of social restrictions.^11^ However, in this study we found no increase in substance misuse related missions (table 2), but rather a decline in the number of intoxicated (≥ 0.5‰) patients encountered by EMS (15.0% vs. 16.2%, *P* < 0.001). This result may be explained by an increased threshold to make emergency calls, but because of the subtle differences between the study groups, caution must be applied when interpreting these results. Last, the proportion of mental health calls increased by 1.2% during the study period (table 2). This finding further supports the hypothesis that there is an association between mental health problems and the COVID-19 pandemic,^18^ possibly due to the social restrictions and global fear of a novel, potentially dangerous infectious disease. Especially, worrisome fact was the decline of median age among mental health patients (41.4 vs. 38.0, *P* = 0.017) during pandemic. Overall, the presented data suggest that in addition to the patients with the actual infection, the challenge among the next COVID-19 waves and other pandemics may also be the various deleterious effects of the social restrictions and lockdowns.

### Limitations

The main limitation of this study covers the selection of the control group. The control group was composed from missions between the years 2016–19 during corresponding months as in the study period. The main benefit of this approach is to reduce short-term randomness in the measurable statistics and control the seasonal fluctuation. However, the total number of EMS missions has been growing steadily during the past years.^19^ Similarly, there may be some factors affecting the change in dispatch codes and patient characteristics, such as the increasing median age of EMS patients. Therefore, we were not able to reliably eliminate these possible confounding factors, and there may be several other factors affecting the observed findings than the COVID-19 pandemic. Despite this bias, the main finding concerning the reduced number of EMS missions during the COVID-19 pandemic remains. If we extrapolate the yearly growth seen in the number of missions in the original data, the decrease is even higher (13–18%; Supplementary figure S1).

## Conclusions

Our study shows a substantial decrease in the number of EMS missions and prolonged time intervals in the study area after the beginning of the COVID-19 pandemic and the declaration of a state of emergency. EMS encountered only a few patients with confirmed COVID-19 infection. These results suggest that the COVID-19 pandemic and social restrictions may play a significant effect on the need for EMS. There is an indispensable need of further development of EMS protocols and strategies for preparation to oncoming waves of the COVID-19 and other novel pandemics.

## Supplementary data

Supplementary data are available at *EURPUB* online.

## Supplementary Material

ckab065_Supplementary_Data

## References

[1] World Health Organization. Rolling updates on coronavirus disease (COVID-19). Available at: https://www.who.int/emergencies/diseases/novel-coronavirus-2019/events-as-they-happen (13 March 2021, date last accessed).

[2] Li H , Liu S, Yu X, et al Coronavirus disease 2019 (COVID-19): current status and future perspectives. Int J Antimicrob Agents 2020;55:105951. 32234466 10.1016/j.ijantimicag.2020.105951 PMC7139247

[3] Thornton J. Covid-19: A&E visits in England fall by 25% in week after lockdown . BMJ 2020;369:m1401. 32253175 10.1136/bmj.m1401

[4] Cao Y , Li Q, Chen J, et al Hospital emergency management plan during the COVID-19 epidemic. Acad Emerg Med 2020;27:309–11. 32124506 10.1111/acem.13951 PMC7159322

[5] Thu TPB , Ngoc PNH, Hai NM, et al Effect of the social distancing measures on the spread of COVID-19 in 10 highly infected countries. Sci Total Environ 2020;742:140430. 32623158 10.1016/j.scitotenv.2020.140430 PMC7307990

[6] Dami F , Berthoz V. Lausanne medical dispatch centre’s response to COVID-19. Scand J Trauma Resusc Emerg Med 2020;28:37. 32404141 10.1186/s13049-020-00735-8 PMC7218525

[7] Perlini S , Canevari F, Cortesi S, et al; the COVID19 IRCCS San Matteo Pavia Task Force. Emergency Department and Out-of-Hospital Emergency System (112-AREU 118) integrated response to Coronavirus Disease 2019 in a Northern Italy centre. Intern Emerg Med 2020;15:825–33. 32507926 10.1007/s11739-020-02390-4 PMC7276336

[8] Finnish Institute for Health and Welfare (THL). COVID-19 cases in the infectious diseases registry. Available at: https://sampo.thl.fi/pivot/prod/fi/epirapo/covid19case/fact_epirapo_covid19case (27 June 2020, date last accessed).

[9] Pekanoja S , Hoikka M, Kyngas H, et al Non-transport emergency medical service missions – a retrospective study based on medical charts. Acta Anaesthesiol Scand 2018;62:701–8. 29363100 10.1111/aas.13071

[10] Vanni G , Legramante JM, Pellicciaro M, et al Effect of lockdown in surgical emergency accesses: experience of a COVID-19 Hospital. In Vivo 2020;34:3033–8. 32871849 10.21873/invivo.12137 PMC7652486

[11] Slavova S , Rock P, Bush HM, et al Signal of increased opioid overdose during COVID-19 from emergency medical services data. Drug Alcohol Depend 2020;214:108176. 32717504 10.1016/j.drugalcdep.2020.108176 PMC7351024

[12] Aitavaara-Anttila M , Liisanantti J, Ehrola A, et al Use of prehospital emergency medical services according to income of residential area. Emerg Med J 2020;37:429–33. 32245748 10.1136/emermed-2019-208834

[13] Holmes JL , Brake S, Docherty M, et al Emergency ambulance services for heart attack and stroke during UK’s COVID-19 lockdown. Lancet 2020;395: e93–4. 32416787 10.1016/S0140-6736(20)31031-X PMC7255139

[14] Bersano A , Kraemer M, Touzé E, et al Stroke care during the COVID-19 pandemic: experience from three large European countries. Eur J Neurol 2020;27:1794–800. 10.1111/ene.14375. 32492764 10.1111/ene.14375 PMC7300856

[15] Mahmood SU , Crimbly F, Khan S, et al Strategies for rational use of personal protective equipment (PPE) among healthcare providers during the COVID-19 crisis. Cureus 2020;12:e8248. 32596068 10.7759/cureus.8248 PMC7308904

[16] European Resuscitation Council COVID-19 Guidelines. Risks Associated With Cardiopulmonary Resuscitation (CPR) in Patients With COVID-19. Niel: ERC, 2020.

[17] The Lancet Gastroenterology & Hepatology. Drinking alone: COVID-19, lockdown, and alcohol-related harm. Lancet Gastroenterol Hepatol 2020; 5:625. 32553138 10.1016/S2468-1253(20)30159-X PMC7295462

[18] Czeisler MÉ , Lane RI, Petrosky E, et al Mental health, substance use, and suicidal ideation during the COVID-19 pandemic - United States, June 24-30, 2020. MMWR Morb Mortal Wkly Rep 2020;69:1049–57. 32790653 10.15585/mmwr.mm6932a1 PMC7440121

[19] Andrew E , Nehme Z, Cameron P, et al Drivers of increasing emergency ambulance demand. Prehosp Emerg Care 2020;24:385. 31237460 10.1080/10903127.2019.1635670

