# Supplementary material for: Early impact of the COVID-19 pandemic and social restrictions on ambulance missions
Source: Eur J Public Health. 2021 Apr 15;31(5):1090–5. doi: 10.1093/eurpub/ckab065 (PMC8083286; doi:10.1093/eurpub/ckab065)
Supplement: ckab065_Supplementary_Data [file ckab065_supplementary_data.zip › ejph-2020-10-om-1246-File004.docx]

**Supplementary Table 1. Emergency Powers Act.**

The Finnish Government’s announcement of the Emergency Powers Act:

• Closure of schools, educational institutions, universities, and civic education (except pre-primary education for children whose parents work in sections critical to the functional society).

• Restriction of public gatherings up to ten persons, and recommendation to avoid unnecessary stay in all public places.

• Closure of museums, theatres, libraries, and other public educational and cultural services.

• Prohibition of visitors to health care units and hospitals.

• High recommendation of quarantine-like conditions for persons over 70 years of age.

• Closure of Finland’s national borders for passenger transportation.

*Specific details available (English)*: https://valtioneuvosto.fi/en/-/10616/hallitus-totesi-suomen-olevan-poikkeusoloissa-koronavirustilanteen-vuoksi
