# Supplementary figures and images for: Early impact of the COVID-19 pandemic and social restrictions on ambulance missions
Source: Eur J Public Health. 2021 Apr 15;31(5):1090–5. doi: 10.1093/eurpub/ckab065 (PMC8083286; doi:10.1093/eurpub/ckab065)

**Supplementary Figure 1. Number of EMS missions (5-day-average) in years 2016-2019 compared to COVID-19 period.**

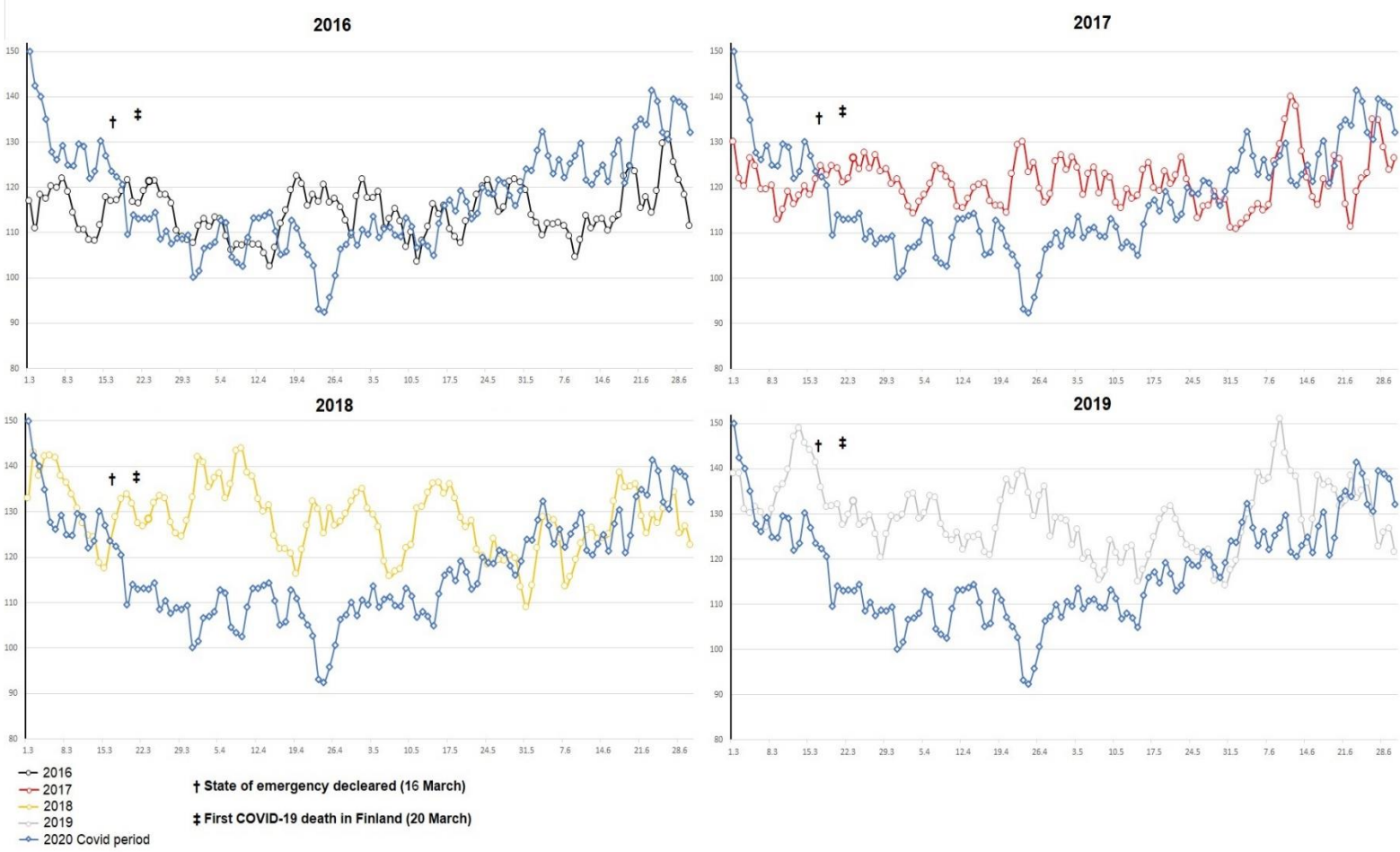

Supplement: ckab065_Supplementary_Data [file ckab065_supplementary_data.zip › ejph-2020-10-om-1246-File003.pdf]
